# Proceedings from the Neurotherapeutics Symposium on Neurological Emergencies: Shaping the Future of Neurocritical Care

**DOI:** 10.1007/s12028-020-01085-0

**Published:** 2020-09-21

**Authors:** Alexis N. Simpkins, Katharina M. Busl, Edilberto Amorim, Carolina Barnett-Tapia, Mackenzie C. Cervenka, Monica B. Dhakar, Mark R. Etherton, Celia Fung, Robert Griggs, Robert G. Holloway, Adam G. Kelly, Imad R. Khan, Karlo J. Lizarraga, Hannah G. Madagan, Chidinma L. Onweni, Humberto Mestre, Alejandro A. Rabinstein, Clio Rubinos, Dawling A. Dionisio-Santos, Teddy S. Youn, Lisa H. Merck, Carolina B. Maciel, the Neurotherapeutics Symposium 2019, Edilberto Amorim, Carolina Barnett-Tapia, Jeremy Brown, Katharina M. Busl, Mackenzie Cervenka, Jan Claassen, Monica Dhakar, Layne Dylla, Marie-Carmelle Elie, Mark Etherton, Kevin Fiscella, Brandon Foreman, Peter Forgacs, Robert C. Griggs, Marc Halterman, Andrea Harriott, Lawrence J. Hirsch, Sara Hocker, Robert Holloway, Rebecca Jules, Adam G. Kelly, Daniel Lackland, Mackenzie P. Lerario, Karlo J. Lizarraga, Carolina B. Maciel, Lisa H. Merck, Gordon Mitchell, Laura B. Ngwenya, Raul G. Nogueira, Clifford Pierre, Javier Provencio, Alejandro A. Rabenstein, Debra Roberts, Clio Rubinos, Eugene Scharf, Kevin N. Sheth, Alexis N. Simpkins, Cleopatra Thurman, Lauren Ullrich, Christopher Zammit

**Affiliations:** 1 Department of Neurology, McKnight Brain Institute, University of Florida College of Medicine, Room L3-100, 1149 Newell Drive, Gainesville, FL 32611 USA; 2 Department of Neurosurgery, University of Florida College of Medicine, Gainesville, FL USA; 3 Department of Neurology, University of California, San Francisco, San Francisco, CA USA; 4 Ellen and Martin Prosserman Centre for Neuromuscular Disorders, Toronto General Hospital, Toronto, ON Canada; 5 Department of Neurology, Johns Hopkins University School of Medicine, Baltimore, MD USA; 6 Department of Neurology, Emory University School of Medicine, Atlanta, GA USA; 7 Department of Neurology, Yale University School of Medicine, New Haven, CT USA; 8 J. Phillip Kistler Stroke Research Center, Massachusetts General Hospital, Boston, MA USA; 9 Department of Neurology, University of Rochester Medical Center, Rochester, NY USA; 10 Department of Critical Care Medicine, Mayo Clinic, Jacksonville, FL USA; 11 Center for Translational Neuromedicine, Department of Neurosurgery, University of Rochester Medical Center, Rochester, USA; 12 Department of Neurology, Mayo Clinic, Rochester, MN USA; 13 Department of Neurology, University of North Carolina School of Medicine, Chapel Hill, NC USA; 14 Department of Neurology, Barrow Neurological Institute, Phoenix, AZ USA; 15 Department of Emergency Medicine, University of Florida College of Medicine, Gainesville, FL USA; 16 Department of Health Outcomes and Biomedical Informatics, University of Florida College of Medicine, Gainesville, FL USA; 17 Department of Neurology, University of Utah, Salt Lake City, UT USA

**Keywords:** Conference proceedings, Diversity excellence, Team science, Translational research, Neurocritical care

## Abstract

Effective treatment options for patients with life-threatening neurological disorders are limited. To address this unmet need, high-impact translational research is essential for the advancement and development of novel therapeutic approaches in neurocritical care. “The Neurotherapeutics Symposium 2019—Neurological Emergencies” conference, held in Rochester, New York, in June 2019, was designed to accelerate translation of neurocritical care research via transdisciplinary team science and diversity enhancement. Diversity excellence in the neuroscience workforce brings innovative and creative perspectives, and team science broadens the scientific approach by incorporating views from multiple stakeholders. Both are essential components needed to address complex scientific questions. Under represented minorities and women were involved in the organization of the conference and accounted for 30–40% of speakers, moderators, and attendees. Participants represented a diverse group of stakeholders committed to translational research. Topics discussed at the conference included acute ischemic and hemorrhagic strokes, neurogenic respiratory dysregulation, seizures and status epilepticus, brain telemetry, neuroprognostication, disorders of consciousness, and multimodal monitoring. In these proceedings, we summarize the topics covered at the conference and suggest the groundwork for future high-yield research in neurologic emergencies.

## Introduction

High-impact translational research is essential for the development of novel therapeutic approaches in neurocritical care. Although it is well known that diversity improves productivity in team science [1–4], there is still a prominent imbalance in the representation of diverse backgrounds and gender in the neuroscience workforce [5–7] and in scientific conferences [6, 8]. “The Neurotherapeutics Symposium 2019—Neurological Emergencies” was designed to address these challenges by creating a platform to facilitate the discussion of innovative translational approaches in acute neurologic illnesses, while specifically seeking the participation of women and under represented minorities in the conference planning and development processes. This 2-day conference was co-hosted by the University of Florida and University of Rochester and was funded by NINDS (R13 NS11956-01), the McKnight Brain Institute, and industry (detailed support is listed under funding section); no funding party provided input in the development of scientific content of the conference or in the proceedings. The symposium was endorsed by the Neurocritical Care Society and adds to the efforts promoting inclusion in the Neurocritical Care Research Network conferences [9]. A total of 90 participants from 25 institutions attended the conference, including 30 from under represented groups, and 42 women. The conference was comprised of 16 lectures, 6 discussion sessions, 2 workshops, a guided poster session, and 4 networking dinner/lunch gatherings. Each lecture was followed by interactive group sessions termed “Looking into the Future,” which sourced participants for next steps in the translational process and were facilitated by pairs of early and established investigators. Opportunities for mentoring and networking between scientists at various career stages were weaved into the curriculum throughout the conference. Digital technology, using an electronic conference ‘app’, also promoted networking among attendees, while hosting a digital repository of e-posters, online discussions, and links to relevant manuscripts.

Another hallmark of the conference was the transdisciplinary participation of scientists from diverse backgrounds, including clinician-scientists, neuroscientists, bioengineers, rehabilitation experts, and trainees. Discussion topics within the conference included: early career development, funding strategies, diversity/team building, epilepsy and status epilepticus, neurotrauma, hypoxic pre-conditioning and neuroplasticity, cerebral edema and secondary brain injury, precision medicine in neurocritical care, neuroprognostication, advanced signal processing of neurophysiologic tests, disorders of consciousness, and neurogenic respiratory dysregulation.

We believe that facilitating such collaborative research has the potential to contribute to the development of long-term networking resources that nurture diversity in team science. Targeted fostering has shown initial success in an example of a women science database [10]. The talent represented by the high proportion of participants in this symposium from under represented groups, extended an opportunity to foster long-lasting mentorship relationships and career building for early career investigators. This is especially important as data support a persistent gap in racial and ethnic representation in the medical education pipeline [11]. Further, while the number of scholars of under represented background has grown, reflecting the establishment of an effective pipeline, attrition throughout their careers remains a significant factor that challenges the sustainability of inclusion efforts; this is supported by the lower proportion of grant funding and awards among these groups [7, 12, 13]. Our goals were aligned with the Neurocritical Care Society’s stated goals for Diversity, Equity & Inclusion [14].

In addition to reviewing the discussed content, we provide a blueprint to shape the future directions of neurocritical care research focused on novel therapeutics. An executive summary of the conference proceedings is shown in Table 1. In the following sections, we summarize the key topics discussed in the conference, including disease-specific future directions for collaborative research.

**Table 1:** Future directions from the Neurotherapeutics Symposium 2019—Neurological Emergencies

Executive summary	
*Strategies for Promoting Success in New Research Initiatives Through Team Science, Diversity, and Novel Funding Mechanisms*	
Intensify efforts in maintaining adequate representation of all stakeholders and backgrounds in future conference planning, speakers and attendees Develop funding mechanisms that promote and facilitate team science approach Expand the use of crowdsourcing funding mechanisms and advertisement of project campaigns across survivors and caregivers groups Leverage the infrastructure provided by Strategies to Innovate Emergency Care Research Network (SIREN) with ancillary (“add-on”) study proposals to major ongoing clinical trials Harmonization of multiple parametric data in future studies and creation of a centralized repository of curated data from clinical trials	
*Stroke and Cerebrovascular Disease*	
Work toward expanding evidence of safety within a broader eligibility criteria for reperfusion therapy in acute ischemic stroke Optimization of systems of care for expeditious triage (imaging and clinical assessments) involving first responders, neurologists, radiologists, neurointerventionalists, and emergency medicine providers Development of adjunctive therapies addressing secondary brain injury mechanisms that can be modulated by interventions targeting spreading depolarization, cerebral edema, white matter tract injury, seizures, and inflammation	
*Therapeutic Intermittent Hypoxia and Neuroplasticity in Neurogenic Respiratory Dysregulation*	
Refinement of acute intermittent hypoxia therapeutic protocols based on the weight of hypoxia depth and burden in competing mechanisms involved in neuroplasticity Combination of acute intermittent hypoxia with pharmacologic modulation of adenosine- and/or serotonin-mediated pathways, with inflammation-targeted strategies Refinement of acute intermittent hypoxia delivery and monitoring methods	
*Brain Telemetry, Seizure Detection and Management in the Critically Ill*	
Optimization of systems of care bypassing the lack of local critical care encephalography expertise, and capitalizing on the use of remote continuous EEG monitoring services Automatic seizure detectors and real-time alarms using quantitative EEG should be developed and validated to expedite timely and accurate review Expansion and refinement of continuous EEG use as well as standardization of algorithms for ischemia monitoring and brain dysfunction to direct individual hemodynamic goals Expansion of status epilepticus international registries to overcome the challenges of examining patients with heterogeneity of disease processes Expansion of antiseizure treatment algorithms to include nutritional therapies (ketogenic diet) and tailored immunomodulation	
*Outcome Prediction in Disorders of Consciousness, Cognition, and Sepsis*	
Objective characterization of gaps of knowledge and methodologic flaws in neuroprognostic studies Unveiling endotypes using multimodality assessment of cerebral reserve and resilience coupled with longitudinal trajectories following critical illness	
*Multimodal Monitoring (MMM) in the Neurocritically Ill*	
Adaptation of strategies to show cost-effectiveness for multimodal monitoring, implement broadly applicable settings where possible, targeting overall clinical performance rather than single datapoints, and integrating various MMM elements and temporal trends Incorporate standardized treatment algorithms, and assess their performance, in studies exploring the value of MMM	

## Strategies for Promoting Success in New Research Initiatives in Neurocritical Care Through Team Science, Diversity, and Novel Funding Mechanisms

The complexities and multifaceted sequela of neurological emergencies require novel strategies for scientific progress and the development of ground breaking therapies with the potential to change outcomes and mitigate disability. In the current era of advanced subspecialties, such as neurocritical care, transdisciplinary teams are critical for health care team functioning [1], as well as scientific discovery and translational research pursuits [2]. Capitalizing on the greater synergistic gains of team science will be an important asset in neurocritical care research. Transdisciplinary team science research often develops in phases, which are summarized in Table 2 [15]. Each phase has its own importance and function in a successful program. The impact of transdisciplinary team science engagement includes cross-disciplinary collaboration, work environments with positive team climate—a perceived set of norms, expectations, and attitudes of a team—[16], and novel approaches to conducting, analyzing, and disseminating research [15–17].

**Table 2:** Phases of Team Science for Translational Research

Phases of Team Science [8, 9]	
Developmental phase—assembling team and developing roles Conceptualization phase—developing a hypotheses framework and scientific approach Implementation phase—conducting the research Translational phase—collaborating to apply results to clinical trial study development	

Transdisciplinary teams include members with resourcefulness, strong delegation skills, diverse backgrounds, and trust in the process of multidisciplinary collaboration. The latter includes the establishment of psychological safety: the notion of standing up to speak on bold concepts, without fear of retaliation or negative repercussions. Diverse teams are also more likely to resolve complex problems [16, 18]. Having team members from diverse backgrounds may be an effective way of altering behaviors of a social majority group and ultimately lead to more accurate group thinking, as diverse groups have an increased tendency to focus on facts and are less likely to make factual errors [19–21]. In addition to bringing more objectivity to the decision-making process and brainstorming activities [20], diverse teams are also more likely to explore novel approaches to problems [21, 22]. Diversity challenges conformity [23], and the resulting innovation and deeper information processing may enrich the potential of scientific progress [24]. Another important aspect of inclusive team science is that diverse faculty members are more likely to engage in research aimed at reducing disparities in neurologic outcomes and spend more time mentoring diverse students and trainees [25, 26]. The Curing Coma Campaign, launched during the Neurocritical Care Society Annual Meeting in 2019, reflects the Neurocritical Care Society efforts in the establishment of longitudinal collaborations in a team science with the mission of addressing disorders of consciousness and promoting awakening [27]. Nurturing diverse, early-career scholars, and trainees from under represented backgrounds is a crucial step to achieve inclusion excellence in neuroscience, as these promising scientists have the potential to become leaders in academic research and inspire future generations to explore innovative pathways to treat neurologic disorders. The Neurocritical Care Society’s Women in Neurocritical Care (WINCC) section demonstrates the commitment toward diversity and equity and is a powerful resource to support early career scholars.

Several studies have demonstrated that knowledge gaps and lack of exposure to racially and ethnically diverse groups, as well as sexual and gender minorities, may lead to barriers in access to clinical care for disadvantaged populations. This may stem from unconscious decisions influenced by the implicit biases of providers [3, 4, 28] or system biases that limit accessibility for diverse populations. Neurodisparity is defined as the effect of disparities in neurologic care; [3] it does not only refer to barriers contributed by race and/or ethnicity, but includes disparities influenced by: sex, gender [7], age, neurodiversity, sexual orientation, religion, geographic origin, disability, weight, incarceration, and other social demographic factors [3]. For example, several publications highlight disparities in representation in the American Academy of Neurology recognition awards and of women in leadership roles in neurology [5, 6]. Neurocritical care, as a burgeoning discipline, is ripe to lead initiatives to increase awareness of knowledge gaps that will address the needs of diverse patient populations and foster adequate representation in clinical leadership and the neuroscience workforce.

Expanding funding mechanisms is essential to support diverse team science efforts [29]. Under represented groups have been reported to receive less support for research funding [13]. Also, the research funds that are provided are more likely to be limited to research topics that pertain to health disparities, disease prevention and intervention, socioeconomic factors, healthcare, lifestyle, psychosocial, adolescence, and risk management [26]. Specifically, funding was less likely to be awarded if these terms were used in comparison with studies that focused on topics linked to neuron, corneal, cell, and iron [26]. Several funding sources were discussed at the conference. Crowdfunding mechanisms are emerging funding opportunities that bring flexibility to support innovative scientific efforts while promoting a platform that bridges investigators and donors; the American Brain Foundation (ABF) Crowdfunding Grant Mechanism (https://www.americanbrainfoundation.org/projects/) is the first neuroscience crowdfunding platform and has received nearly 150 donations and funded 8 projects (as of 2019). The National Institute of Health (NIH) Strategies to Innovate Emergency Care Research Network (SIREN) [30] represents a new network established in 2017 with 11 regional centers and 50 satellite research sites, aimed to develop acute neurological emergency research across the disciplines of Neurology, Neurosurgery, Emergency Medicine and Critical Care, and leverage this infrastructure to support multicenter randomized clinical trials (RCT). This multidisciplinary approach to clinical trials in the study of status epilepticus [31, 32] and stroke [33–35] have led to the identification of successful interventions. The Neurocritical Care Society has supported a growing grant funding portfolio that reflects its transdisciplinary mission and includes the Research Training—a 12-month research fellowship for physicians, nurses, scientists, advanced practitioners, among other specialties—and the INvesting in CLINical Neurocritical CarE Research (INCLINE) grant, which fosters multicenter outcome-focused research. The Neurocritical Care Research Central (NCRC) was developed to streamline research efforts within members of the Neurocritical Care Society. The Neurocritical Care Research Network (NCRN)—a subcommittee of the NCRC—was developed with the goal of bringing together a cohesive group of established researchers to facilitate successful implementation of patient-oriented research.

Further, efforts in harmonizing data collection in future trials of acute brain injuries, including curated multiple parametric and imaging data repositories, are urgently needed to facilitate subsequent data analyses and cohort discovery across populations.

## Disease-Specific Knowledge Gaps and Future Directions

The following represents a summary of topics discussed during the conference. Due to the nature of its format as a live conference with interactive presentations and critical debates, this summary highlights the major points of discussion and perceived future directions of research proposed by the Neurotherapeutics Symposium 2019 attendees.

### Stroke and Cerebrovascular Diseases

Major advances in the treatment of ischemic stroke secondary to large-vessel occlusion have led to improved outcomes [33–35]. Patient selection mediated through advanced neuroimaging has expanded the eligibility of reperfusion therapies for patients outside the conventional treatment windows or with an unknown last-known-well time [36–38]. Novel strategies in stroke emphasize the role of neuroimaging to elucidate contributing mechanisms for neurologic recovery and further guide patient selection for acute therapy.

The current patient selection criteria for endovascular thrombectomy (EVT)—particularly late-window—are relatively strict, which deprives a significant proportion of patients from targeted revascularization [39]. Decision-making in acute therapy relies on the defined inclusion criteria from clinical trials. This may be best epitomized by decisions regarding EVT, which is dictated in large part by factors including time from symptom onset, the presence of preexisting disability, and ischemic core volume estimates. The positive early- and late-windows EVT trials relied on neuroimaging markers for patient selection that varied in complexity from CT head and angiography (MR CLEAN [40]) and target mismatch on perfusion imaging (SWIFT PRIME [41] and DEFUSE-3 [34]). A relatively large ischemic burden reflected by Alberta Stroke Program Early CT Score (ASPECTS) scores of 5 or 6 is often used as a reason to not proceed with EVT within 6 h of last known well, though in some cases this score could reflect injury to areas of the brain that are less eloquent, and therefore, less likely to portend a poor outcome. Other limitations of using imaging as a strict criterion for therapy eligibility include early diffusion weighted image (DWI) reversibility [42–44] and CT perfusion “ghost” core [45]. Similarly, the presence of preexisting disability (e.g., inability to ambulate independently) is often used as an exclusion, yet providers should interpret preexisting disability from the perspective of the individual patient, not as an immutable construct. A paradigm shift was proposed, especially for the early window (< 6 h), where considerations should be made to explore individualized decision-making such that the pool for those whom may benefit can be broadened. The goal would be to continue research efforts to determine which patients not included in published randomized control trials may still benefit from EVT without increasing the risk of harm.

Beyond reperfusion therapies, stroke-related therapeutics must expand to target white matter disease and cytotoxic edema pathways, and provide biologic biomarkers for patient individualized therapies [46, 47]. Premorbid white matter structural injury of presumed vascular origin can be characterized on MRI by diffusion tensor imaging (DTI) and inform functional outcomes [48, 49]. The characterization of white matter structural integrity could allow for a precision medicine-based prognostication and stroke outcome modeling, advancing knowledge on the critical white matter tracts involved in stroke recovery, increasing patient enrichment for clinical trials design, and potentially, leading to the future development of novel therapeutics. There are multiple ongoing early phase clinical trials testing lifestyle, behavior, and pharmacologic interventions aimed at reducing progression of white matter injury and cerebral small vessel disease [50].

Pathways leading to cytotoxic edema after stroke represent another target for novel therapeutics with promising findings in pre-clinical and early clinical trials. Glyburide—an inhibitor of the ATP-sensitive potassium channel regulatory subunit sulfonylurea receptor 1—is an emerging therapy with favorable results on metrics of edema in the phase II RCT of hemispheric strokes (GAMES-RP [51, 52]). A phase III RCT testing the efficacy and safety of glyburide for severe cerebral edema after large hemispheric infarction (CHARM) has commenced enrollment (ClinicalTrials.gov Identifier: NCT02864953). This cytotoxic edema mechanism is probably shared by other acute brain injuries, and its modulation may represent a therapy with a role beyond acute ischemic stroke.

Deepening the understanding of mechanisms underpinning secondary brain injury in cerebrovascular disease is another important step toward neurotherapeutics. Cortical spreading depolarization and depression—self-propagating waves of near complete breakdown of ion gradient homeostasis—is a potential mechanism of secondary injury in traumatic brain injury, acute ischemic stroke, intracerebral hemorrhage, and subarachnoid hemorrhage (SAH) often triggered by extravasation of blood, trauma or focal ischemia [53]; however, a direct causal relationship remains to be demonstrated [54]. While the modulation of spreading depolarization as a potential therapeutic target in cerebrovascular diseases seems feasible given the wide range of available drugs that suppress its occurrence, there are important barriers to be overcome. There are no reliable noninvasive methods for spreading depolarization detection with high temporal resolution, and neurophysiologic invasive monitoring—the gold standard—has limited spatial resolution and requires violation of the cranial vault. In another angle, a novel filtration system (termed neuroapheresis) designed to rapidly remove blood and blood byproducts from cerebral spinal fluid in patients with SAH tackles the hypothesis that blood breakdown products in SAH are detrimental to outcomes. The feasibility study on this system has demonstrated that an automated lumbar catheter system can safely filter cerebral spinal fluid and remove blood byproducts [55], and may be associated with a positive impact in neuroinflammation.

These parallel avenues of translational research seek to improve long-term outcomes after cerebrovascular diseases through novel neurotherapeutics. Harmonizing existing clinical databases and standardizing neuroimaging and neurophysiology interpretations for the purpose of inter-institutional collaborations sharing advanced expertise (such as the application of machine learning to big datasets) will facilitate these efforts [46].

### Intermittent Hypoxia and Neuroplasticity in Neurogenic Respiratory Dysregulation

Chronic respiratory failure is a common and extremely disabling complication of motor neuron disease and cervical spinal cord injury [56], rendering patients dependent on mechanical ventilation and prone to have respiratory tract infections. The mechanisms underlying respiratory motor neuron plasticity and resilience to injury remain poorly understood, hindering advancement in neurotherapeutics focused on respiratory function rehabilitation [57].

Ischemic preconditioning through acute intermittent hypoxia (AIH) is a promising therapy with potential to prevent respiratory deconditioning and enhance rehabilitation in chronic respiratory failure [58, 59]. AIH promotes phrenic nerve long-term facilitation through carotid chemoreceptors and raphe nuclei modulation [57]. Respiratory motor neuron plasticity is mediated by balancing 5-HT receptor activation and adenosine (A2A) receptor antagonism at different levels of hypoxia depth and duration [60, 61]. The induction and maintenance of AIH follow different signaling pathways that allow for long-lasting phrenic motor facilitation to take place. In this context, 5-HT and A2A receptors can have both favorable and deleterious effects for neuron plasticity depending on the degree of activation or antagonism of these receptors. Serotonin can enhance synaptic transmission in motor neurons, while adenosine can be neurotoxic for phrenic motor neurons. Promising preliminary findings in human studies using AIH include improvement in exercise endurance by athletes and respiratory mechanics in patients who are ventilator dependent and had a phrenic diaphragm pacemaker (increase in maximal inspiratory pressure) [59, 62, 63]. Interestingly, increased muscle strength was not seen exclusively in diaphragmatic muscles in patients with chronic spinal cord injury. Forelimb function and walking ability were also improved when AIH was added to standard rehabilitation exercises [64]. These effects were dose dependent and varied according to the fraction of inspired oxygen level and number of AIH cycles per day.

These translational and pilot human studies highlight the complexity of the biological mechanisms involved in neuroplasticity. The high variability in respiratory mechanics improvement depending on AIH methods, and the depth and duration of hypoxic events, support the hypothesis that respiratory motor neuron plasticity relies on balancing several and often competing pathways. These methods can have beneficial, but also pathologic consequences to patients; therefore, the refinement of AIH therapies in future studies is imperative. Biomarkers of short-term and long-term respiratory function will be fundamental to guide the delivery and monitoring of these therapies. Future studies should combine AIH strategies with pharmacological interventions, such as adenosine antagonism with caffeine or autoinflammatory modulation with corticosteroids—an approach with the potential to enhance the already promising results of AIH in motor neuron disease and spinal cord injury.

### Brain Telemetry, Seizure Detection and Management in the Critically Ill

Continuous electroencephalography (cEEG) is an integral part of the evaluation of critically ill patients with encephalopathy as nonconvulsive seizures are common in the critically ill and may negatively impact outcomes [65, 66]. A cross-sectional analysis using the National Inpatient Sample database including over 7 million critically ill patients, 22,728 of whom underwent cEEG, suggested a positive impact of continuous monitoring on mortality in the subset of patients with intracerebral hemorrhage, SAH and those with encephalopathy, which may offset the increased cost of hospitalization associated with monitoring [67]. Recent studies indicate that epileptiform activity on EEG combined with changes in quantitative EEG measures, such as reduced alpha–delta ratio and relative alpha variability, can detect ischemia earlier than conventional modalities in patients with SAH [68, 69]. Despite these emerging benefits of cEEG beyond seizure detection, this tool is still under-utilized. Potential barriers to the expansion of its use as a brain telemetry instrument include the lack of resources for cEEG acquisition equipment and data storage, limited availability of trained EEG technologists, lack of experts in ICU EEG interpretation to provide timely review of the EEG to be meaningful to bedside clinicians, and the variability in interpretation of abnormal findings, and therapeutic management strategies.

Status epilepticus (SE) is a common neurologic emergency. Over the last decade, the role of pro-inflammatory mediators in seizure generation and propagation, and the search for novel therapeutic anti-inflammatory targets have been areas of intense research. Novel tailored immunotherapies targeting specific cytokines (e.g., interleukin-1 receptor antagonist—anakinra, and interleukin-6 receptor inhibitor—tocilizumab) have been shown to be effective in new-onset refractory status epilepticus (NORSE) and febrile-infection related epilepsy syndrome (FIRES) [70, 71]. In addition, the expansion of ketogenic diet to the critical care setting has been demonstrated to be feasible and effective with success rates in weaning anesthetics without recurrence of SE surpassing 70%, even when after 420 days from SE onset [72–74]. While these emerging therapies are promising novel treatment options, there remains a paucity of randomized controlled trials showing definitive safety, feasibility, and efficacy. Challenges that were identified as a first step in moving the needle toward the development of new treatment strategies and conducting clinical trials include: the heterogeneity of SE etiologies, lack of standardization of treatment protocols, and variations in the selected outcomes.

### Outcome Prediction in Disorders of Consciousness, Cognition, and Sepsis

Impairment in consciousness and cognition can occur in the setting of a primary neurologic disease (e.g., hypoxic-ischemic, hemorrhagic, or traumatic brain injury), or as a consequence of a systemic insult (e.g., toxic, metabolic, and/or infectious processes). Invariably, an interplay of multiple factors concurrently contributes to the development of encephalopathy. As a result, predicting the short- and long-term consequences of impaired consciousness and cognition remains a challenge, in which the self-fulfilling prophecy bias of premature pessimistic outcome predictions plays an important role [75, 76]. Key disease-specific knowledge gaps in neuroprognostication have been recently unveiled in a joint statement from the German Neurocritical Care Society and the Neurocritical Care Society [77]; future studies should account for these limitations. Population-derived disease severity scores are incorrectly used to ascertain long-term individual prognostic trajectories. Further, these scores are not dynamic and are frequently inaccurate. Accurate neuroprognostication is crucial to guide critical care resource allocation and to guide families in goals of care discussions; thus, it is imperative to improve tools for prognostication in neurocritically ill patients. cEEG monitoring provides dynamic information on brain physiology, and it can be used as a noninvasive biomarker that correlates with level of consciousness [78] and neurological recovery [79]. Machine learning has been demonstrated to be a promising tool to analyze EEG data and unveil patients with preserved awareness by means of electroencephalographic activation in patients with acute brain injury [78]. This state known as “cognitive motor dissociation” was previously demonstrated in chronically unresponsive patients [80], and more recently in acute brain injury [78, 81]. Similarly, the use of machine learning and quantitative analysis of longitudinal EEG signals shows promise in improving accuracy of commonly employed neurophysiologic predictors of outcome after cardiac arrest, such as background reactivity, epileptiform activity, and background continuity [79, 82].

Our understanding of the pathophysiology behind sepsis-associated encephalopathy (SAE) remains limited and is another area of much needed research in neurocritical care given the long-term disability associated with persistent cognitive deficits [83, 84]. A complex interplay of factors is involved in SAE pathophysiology and includes the release of damage associated molecular pattern (DAMP) molecules as potent activators of the innate immune system leading to the release of neuroinflammatory cytokines, which cause disruption of the brain blood barrier, activation of endothelial cells, mitochondrial dysfunction, cellular hypoxia, impaired brain perfusion regulation, and apoptosis [85]. Methods to quantify the degree of neuronal injury associated with sepsis are urgently needed to characterize the spectrum of SAE, identify potential targeted therapies, and tailor rehabilitation. Chemical biomarkers of neuronal injury, such as serum neuron specific enolase (NSE), S-100 B and neurofilament (Nf), glial fibrillary acidic protein (GFAP), co-peptin, tau, and ubiquitin C-terminal hydrolase L1 (UCH-L1), are currently being investigated to fulfill this role (ClinicalTrials.gov Identifier: NCT03133208). In addition, there might be an association between elevations of certain biomarkers with specific microbiota. Currently, laboratory tests that can be ordered clinically are still not widely available at every institution and often may not result for several days to weeks, limiting its applicability in clinical practice.

## Multimodal Monitoring (MMM) in the Neurocritical Care Unit

Multimodal monitoring in neurocritical care includes, but is not limited to, cEEG, intracranial pressure (ICP) monitoring, and measurement of cerebral blood flow surrogates, cerebral oxygenation, and cerebral microdialysis. The employment of MMM is mainly targeted to detect factors associated with the development of secondary brain injury, often as a result of bioenergetic failure, and monitor responses to targeted therapies. Various MMM subtypes aim to evaluate tissue function by assessing individual measures of metabolism, blood supply, or oxygenation. Further, MMM allows for the reliable detection of spreading depolarizations and their metabolic toll in cerebral tissue. For each parameter being assessed by MMM, there are several drawbacks that limit the expansion of this modality in clinical practice. Targeting individual values from MMM is unlikely to improve clinical outcomes in isolation. The assessment of average values may miss transient changes that, cumulatively, may result in a higher injury burden. The lack of integral data analysis precludes the incorporation of time in relation to the brain injury and the duration of secondary insults. Additionally, not accounting for individual resilience to insults, and the morbidity of certain therapies (e.g., increased sedation or the use of vasopressors for hemodynamic augmentation), challenges the ability of MMM to demonstrate a positive impact in clinical outcomes.

## Conclusions

The Neurotherapeutics Symposium 2019 included a transdisciplinary participation of scholars from diverse backgrounds, research interest and fields, and subspecialty focus. The conference highlighted the perceived clinically relevant research needs in various acute brain injuries and outlined future strategies on how to expand upon them while incorporating diversity and team science. We hope this format will also encourage continued mentorship and career building for early career investigators to help support and sustain a forward feeding pipeline of women and under represented minorities to advance translational research in neurologic emergencies. A focus on strong research networks, streamlined data sharing practices, and support for early investigators will increase the likelihood of important discoveries.

## References

[1] Fiscella K, Mauksch L, Bodenheimer T, Salas E (2017). Improving care teams’ functioning: recommendations from team science. Jt Comm J Qual Patient Saf.

[2] Bennett LM, Gadlin H (2012). Collaboration and team science: from theory to practice. J Investig Med.

[3] Marulanda-Londono ET, Bell MW, Hope OA (2019). Reducing neurodisparity: recommendations of the 2017 AAN diversity leadership program. Neurology.

[4] Rosendale N, Ostendorf T, Evans DA (2019). American academy of neurology members’ preparedness to treat sexual and gender minorities. Neurology.

[5] Silver JK (2019). Understanding and addressing gender equity for women in neurology. Neurology.

[6] Fournier LE, Hopping GC, Zhu L (2020). Females are less likely invited speakers to the international stroke conference: time’s up to address sex disparity. Stroke.

[7] Silver JK, Bank AM, Slocum CS (2018). Women physicians underrepresented in American Academy of Neurology recognition awards. Neurology.

[8] Ruzycki SM, Fletcher S, Earp M, Bharwani A, Lithgow KC (2019). Trends in the proportion of female speakers at medical conferences in the United States and in Canada, 2007 to 2017. JAMA Netw Open.

[9] Hocker S, Shah S, Vespa P (2020). The future of neurocritical care research: proceedings and recommendations from the fifth neurocritical care research network conference. Neurocrit Care.

[10] McCullagh EA, Nowak K, Pogoriler A, Metcalf JL, Zaringhalam M, Zelikova TJ (2019). Request a woman scientist: a database for diversifying the public face of science. PLoS Biol.

[11] Lett LA, Murdock HM, Orji WU, Aysola J, Sebro R (2019). Trends in racial/ethnic representation among US medical students. JAMA Netw Open.

[12] Witteman HO, Hendricks M, Straus S, Tannenbaum C (2019). Are gender gaps due to evaluations of the applicant or the science? A natural experiment at a national funding agency. Lancet.

[13] Ginther DK, Schaffer WT, Schnell J (2011). Race, ethnicity, and NIH research awards. Science.

[14] Diversity, Equity and Inclusion. Neurocritical Care Society. https://www.neurocriticalcare.org/about/dei. Accessed 09 July 2020.

[15] Hall KL, Vogel AL, Stipelman B, Stokols D, Morgan G, Gehlert S (2012). A four-phase model of transdisciplinary team-based research: goals, team processes, and strategies. Transl Behav Med.

[16] Settles IH, Brassel ST, Soranno PA, Cheruvelil KS, Montgomery GM, Elliott KC (2019). Team climate mediates the effect of diversity on environmental science team satisfaction and data sharing. PLoS ONE.

[17] Vogel AL, Stipelman BA, Hall KL, Nebeling L, Stokols D, Spruijt-Metz D (2014). Pioneering the transdisciplinary team science approach: lessons learned from national cancer institute grantees. J Transl Med Epidemiol.

[18] Gomez LE, Bernet P (2019). Diversity improves performance and outcomes. J Natl Med Assoc.

[19] Levine SS, Apfelbaum EP, Bernard M, Bartelt VL, Zajac EJ, Stark D (2014). Ethnic diversity deflates price bubbles. Proc Natl Acad Sci USA.

[20] Sommers SR (2006). On racial diversity and group decision making: identifying multiple effects of racial composition on jury deliberations. J Pers Soc Psychol.

[21] Phillips KW, Liljenquist KA, Neale MA (2009). Is the pain worth the gain? The advantages and liabilities of agreeing with socially distinct newcomers. Pers Soc Psychol Bull.

[22] Nathan M, Lee N (2013). Cultural diversity, innovation, and entrepreneurship: firm-level evidence from London. Econ Geogr.

[23] Talke K, Salomo S, Kock A (2011). Top management team diversity and strategic innovation orientation: the relationship and consequences for innovativeness and performance. J Prod Innov Manag.

[24] Galinsky AD, Todd AR, Homan AC (2015). Maximizing the gains and minimizing the pains of diversity: a policy perspective. Perspect Psychol Sci.

[25] de Dios MA, Kuo C, Hernandez L (2013). The development of a diversity mentoring program for faculty and trainees: a program at the Brown Clinical Psychology Training Consortium. Behav Ther (NY).

[26] Hoppe TA, Litovitz A, Willis KA (2019). Topic choice contributes to the lower rate of NIH awards to African-American/black scientists. Sci Adv.

[27] Provencio JJ, Hemphill JC, Claassen J, et al. The curing coma campaign: framing initial scientific challenges-proceedings of the first curing coma campaign scientific advisory council meeting. In: Neurocritical Care; 2020. 10.1007/s12028-020-01028-9 PMC7392933 32578124

[28] Pritlove C, Juando-Prats C, Ala-Leppilampi K, Parsons JA (2019). The good, the bad, and the ugly of implicit bias. Lancet.

[29] Carnethon MR, Kershaw KN, Kandula NR (2019). Disparities research, disparities researchers, and health equity. JAMA.

[30] Beam DM, Brown J, Kaji AH (2020). Evolution of the strategies to innovate emergency care clinical trials network (SIREN). Ann Emerg Med.

[31] Silbergleit R, Durkalski V, Lowenstein D (2012). Intramuscular versus intravenous therapy for prehospital status epilepticus. N Engl J Med.

[32] Kapur J, Elm J, Chamberlain JM (2019). Randomized trial of three anticonvulsant medications for status epilepticus. N Engl J Med.

[33] Nogueira RG, Jadhav AP, Haussen DC (2018). Thrombectomy 6 to 24 hours after stroke with a mismatch between deficit and infarct. N Engl J Med.

[34] Albers GW, Marks MP, Kemp S (2018). Thrombectomy for stroke at 6 to 16 hours with selection by perfusion imaging. N Engl J Med.

[35] Goyal M, Menon BK, van Zwam WH (2016). Endovascular thrombectomy after large-vessel ischaemic stroke: a meta-analysis of individual patient data from five randomised trials. Lancet.

[36] Schwamm LH, Wu O, Song SS, et al. IV Alteplase in MR-selected patients with stroke of unknown onset is safe and feasible: results of the multicenter MR WITNESS Trial (NCT01282242). In: International stroke conference; 17–19 February 2016; Los Angeles, CA.

[37] Thomalla G, Simonsen CZ, Boutitie F (2018). MRI-guided thrombolysis for stroke with unknown time of onset. N Engl J Med.

[38] Ma H, Campbell BCV, Parsons MW (2019). Thrombolysis guided by perfusion imaging up to 9 hours after onset of stroke. N Engl J Med.

[39] Nogueira RG, Ribo M (2019). Endovascular treatment of acute stroke. Stroke.

[40] Berkhemer OA, Fransen PS, Beumer D (2015). A randomized trial of intraarterial treatment for acute ischemic stroke. N Engl J Med.

[41] Saver JL, Goyal M, Bonafe A (2015). Stent-retriever thrombectomy after intravenous t-PA vs. t-PA alone in stroke. N Engl J Med.

[42] Simpkins AN, Dias C, Norato G, Kim E, Leigh R, Investigators NIHNHoS (2017). Early change in stroke size performs best in predicting response to therapy. Cerebrovasc Dis.

[43] Naqvi I, Simpkins AN, Cullison K (2018). Recurrent thrombolysis of a stuttering lacunar infarction captured on serial MRIs. eNeurologicalSci.

[44] Tahsili-Fahadan P, Simpkins AN, Leigh R, Merino JG (2016). Stuttering lacunar infarction captured on serial MRIs. Neurol Clin Pract.

[45] Kim-Tenser M, Mlynash M, Lansberg MG, et al. CT perfusion core and ASPECT score prediction of outcomes in DEFUSE 3. Int J Stroke. 2020;1747493020915141. 10.1177/1747493020915141. 10.1177/1747493020915141 32233746

[46] Simpkins AN, Janowski M, Oz HS (2020). Biomarker application for precision medicine in stroke. Transl Stroke Res.

[47] Nadareishvili Z, Kelley D, Luby M (2019). Molecular signature of penumbra in acute ischemic stroke: a pilot transcriptomics study. Ann Clin Transl Neurol.

[48] Etherton MR, Wu O, Cougo P (2017). Integrity of normal-appearing white matter and functional outcomes after acute ischemic stroke. Neurology.

[49] Etherton MR, Wu O, Giese AK (2019). White matter integrity and early outcomes after acute ischemic stroke. Transl Stroke Res.

[50] Smith EE, Markus HS (2020). New treatment approaches to modify the course of cerebral small vessel diseases. Stroke.

[51] Kimberly WT, Bevers MB, von Kummer R (2018). Effect of IV glyburide on adjudicated edema endpoints in the GAMES-RP Trial. Neurology.

[52] Sheth KN, Elm JJ, Molyneaux BJ (2016). Safety and efficacy of intravenous glyburide on brain swelling after large hemispheric infarction (GAMES-RP): a randomised, double-blind, placebo-controlled phase 2 trial. Lancet Neurol.

[53] Shuttleworth CW, Andrew RD, Akbari Y (2020). Which spreading depolarizations are deleterious to brain tissue?. Neurocrit Care.

[54] Helbok R, Hartings JA, Schiefecker A (2020). What should a clinician do when spreading depolarizations are observed in a patient?. Neurocrit Care.

[55] Blackburn SL, Grande AW, Swisher CB, Hauck EF, Jagadeesan B, Provencio JJ (2019). Prospective trial of cerebrospinal fluid filtration after aneurysmal subarachnoid hemorrhage via lumbar catheter (PILLAR). Stroke.

[56] Navarrete-Opazo A, Mitchell GS (2014). Therapeutic potential of intermittent hypoxia: a matter of dose. Am J Physiol Regul Integr Comp Physiol.

[57] Seven YB, Mitchell GS (2019). Mechanisms of compensatory plasticity for respiratory motor neuron death. Respir Physiol Neurobiol.

[58] Trumbower RD, Jayaraman A, Mitchell GS, Rymer WZ (2012). Exposure to acute intermittent hypoxia augments somatic motor function in humans with incomplete spinal cord injury. Neurorehabil Neural Repair.

[59] Foster GE, Brugniaux JV, Pialoux V (2009). Cardiovascular and cerebrovascular responses to acute hypoxia following exposure to intermittent hypoxia in healthy humans. J Physiol.

[60] Seven YB, Simon AK, Sajjadi E, Zwick A, Satriotomo I, Mitchell GS (2020). Adenosine 2A receptor inhibition protects phrenic motor neurons from cell death induced by protein synthesis inhibition. Exp Neurol.

[61] Tadjalli A, Mitchell GS (1985). Cervical spinal 5-HT2A and 5-HT2B receptors are both necessary for moderate acute intermittent hypoxia-induced phrenic long-term facilitation. J Appl Physiol.

[62] Lovett-Barr MR, Satriotomo I, Muir GD (2012). Repetitive intermittent hypoxia induces respiratory and somatic motor recovery after chronic cervical spinal injury. J Neurosci.

[63] Navarrete-Opazo A, Vinit S, Dougherty BJ, Mitchell GS (2015). Daily acute intermittent hypoxia elicits functional recovery of diaphragm and inspiratory intercostal muscle activity after acute cervical spinal injury. Exp Neurol.

[64] Hayes HB, Jayaraman A, Herrmann M, Mitchell GS, Rymer WZ, Trumbower RD (2014). Daily intermittent hypoxia enhances walking after chronic spinal cord injury: a randomized trial. Neurology.

[65] Claassen J, Taccone FS, Horn P (2013). Recommendations on the use of EEG monitoring in critically ill patients: consensus statement from the neurointensive care section of the ESICM. Intensive Care Med.

[66] Herman ST, Abend NS, Bleck TP (2015). Consensus statement on continuous EEG in critically ill adults and children, part I: indications. J Clin Neurophysiol.

[67] Hill CE, Blank LJ, Thibault D (2019). Continuous EEG is associated with favorable hospitalization outcomes for critically ill patients. Neurology.

[68] Rots ML, van Putten MJ, Hoedemaekers CW, Horn J (2016). Continuous EEG monitoring for early detection of delayed cerebral ischemia in subarachnoid hemorrhage: a pilot study. Neurocrit Care.

[69] Rosenthal ES, Biswal S, Zafar SF (2018). Continuous electroencephalography predicts delayed cerebral ischemia after subarachnoid hemorrhage: a prospective study of diagnostic accuracy. Ann Neurol.

[70] Kenney-Jung DL, Vezzani A, Kahoud RJ (2016). Febrile infection-related epilepsy syndrome treated with anakinra. Ann Neurol.

[71] Jun JS, Lee ST, Kim R, Chu K, Lee SK (2018). Tocilizumab treatment for new onset refractory status epilepticus. Ann Neurol.

[72] Park EG, Lee J, Lee J (2019). The ketogenic diet for super-refractory status epilepticus patients in intensive care units. Brain Dev.

[73] Francis BA, Fillenworth J, Gorelick P, Karanec K, Tanner A (2019). The feasibility, safety and effectiveness of a ketogenic diet for refractory status epilepticus in adults in the intensive care unit. Neurocrit Care.

[74] Cervenka MC, Hocker S, Koenig M (2017). Phase I/II multicenter ketogenic diet study for adult superrefractory status epilepticus. Neurology.

[75] Geocadin RG, Callaway CW, Fink EL (2019). Standards for studies of neurological prognostication in comatose survivors of cardiac arrest: a scientific statement from the American Heart Association. Circulation.

[76] McCracken DJ, Lovasik BP, McCracken CE (2019). The intracerebral hemorrhage score: a self-fulfilling prophecy?. Neurosurgery.

[77] Wartenberg KE, Hwang DY, Haeusler KG (2019). Gap analysis regarding prognostication in neurocritical care: a joint statement from the german neurocritical care society and the neurocritical care society. Neurocrit Care.

[78] Claassen J, Doyle K, Matory A (2019). Detection of brain activation in unresponsive patients with acute brain injury. N Engl J Med.

[79] Amorim E, van der Stoel M, Nagaraj SB (2019). Quantitative EEG reactivity and machine learning for prognostication in hypoxic-ischemic brain injury. Clin Neurophysiol.

[80] Kondziella D, Friberg CK, Frokjaer VG, Fabricius M, Moller K (2016). Preserved consciousness in vegetative and minimal conscious states: systematic review and meta-analysis. J Neurol Neurosurg Psychiatry.

[81] Edlow BL, Chatelle C, Spencer CA (2017). Early detection of consciousness in patients with acute severe traumatic brain injury. Brain.

[82] Tjepkema-Cloostermans MC, da Silva Lourenco C, Ruijter BJ (2019). Outcome prediction in postanoxic coma with deep learning. Crit Care Med.

[83] Feng Q, Ai YH, Gong H (2019). Characterization of sepsis and sepsis-associated encephalopathy. J Intensive Care Med.

[84] Pandharipande PP, Girard TD, Jackson JC (2013). Long-term cognitive impairment after critical illness. N Engl J Med.

[85] Rajaee A, Barnett R, Cheadle WG (2018). Pathogen- and danger-associated molecular patterns and the cytokine response in sepsis. Surg Infect (Larchmt).

